# Plant Uptake and Distribution of Endosulfan and Its Sulfate Metabolite Persisted in Soil

**DOI:** 10.1371/journal.pone.0141728

**Published:** 2015-11-03

**Authors:** Jeong-In Hwang, Sung-Eun Lee, Jang-Eok Kim

**Affiliations:** School of Applied Biosciences, Kyungpook National University, Daegu, Republic of Korea; Yeungnam University, REPUBLIC OF KOREA

## Abstract

The distributions of endosulfan (ED) residues (α-, β-isomers, and sulfate-metabolite) in cucumbers grown in soils treated with ED at concentrations of 20 and 40 mg kg^-1^ were assessed using indoor and outdoor experiments. In all treatments, degradation rates of the α-isomer in soils were higher than that of the β-isomer. In the indoor tests, uptake amounts of total ED by cucumbers, after 15 d of growth, were 7.8 and 14.5 mg kg^-1^ in 20 and 40 mg kg^-1^-treated pots, respectively. For growth time from 15 to 30 d, uptake amounts in 20 and 40 mg kg^-1^-treated pots were 3.8 and 7.9 mg kg^-1^, respectively. Outdoor tests resulted in smaller ED residues in cucumbers than those in indoor tests. In both indoor and outdoor tests, ED residues absorbed were highest in roots, and the α-isomer was the more frequently absorbed isomer. These results will be useful for determining management criteria for soil persistent pesticides.

## Introduction

Several recent studies have reported pesticide residues in organic agricultural products [1,2]. The translocation and persistence of pesticides absorbed from agricultural soils into crops is undesirable as it can result in safety issues in the final agricultural products [3,4]. However, most studies have focused on the detection of residues in edible parts following pesticide application [5,6], and information on plant uptake patterns of soil persistent pesticides is largely lacking.

The pesticide uptake pattern of plants may depend on the application concentration and degradation of the pesticides [7,8]. For example, uptake patterns of chlorpyrifos by potatoes corresponded closely to its degradation patterns in soil under conditions of consecutive application [9]. Plant growth, which may be responsible for diluting absorbed pesticides during growth, may also influence the uptake patterns. Anderson *et al*. [10] reported that cyantraniliprole absorbed by tomatoes at the first stage of growth decreased by 55.5% in leaves and fruits as growth progressed. Therefore, it is required to monitor plant growth parameters, such as weight and length, when determining the uptake and distribution of pesticides.

Organochlorine insecticide, endosulfan (6,7,8,9,10-hexachloro-1,5,5a,6,9,9,a-hexahydro-6,9-methano-2,3,4-benzodioxathiepin-3-oxide, ED) consisted of α- and β-isomers in the ratio of 7:3 and has been globally used to control a number of pests [11]. However, ED persists for long periods in the soil and forms a metabolite, ED-sulfate, which is more toxic and persists longer than the parent compound (α- and β-isomers) [11]. The ED-sulfate is particularly stable with a half-life of approximately 100 days in soil, whereas ED has its half-life of approximately 43 d in soil [12,13]. Therefore, the uptake of considerable amounts of ED-sulfate from agricultural soils by crops can be increased by the uptake of ED. Although many studies have reported the persistence and degradation of the ED and its metabolite in the soil, studies regarding their uptake and distribution from soils to crops are sparse.

In the present study, cucumbers were used to assess the dynamic uptake patterns of ED from soils into plants. Uptake studies were conducted under both indoor and outdoor conditions, and residual amounts of the ED were analyzed in four plant compartments, including roots, stems, leaves, and fruits. The analysis data for ED residues were recalculated with varying levels of ED exposure concentration and degradation patterns as well as various stages of plant growth.

## Materials and Methods

### Chemicals

Analytical standards of α- and β-ED and ED-sulfate were purchased from Riedel-de Haёn^®^ (Deisenhofen, Germany). Commercial ED as emulsifiable concentrate (EC, 35%) was obtained from Dongbu Farm Hannong (Seoul, Republic of Korea). Florisil (F0127, 60–100 mesh) was purchased from Sigma-Aldrich Chemical Co. (St. Louis, MO, USA) and acetone, methylene chloride, *n*-hexane, and ethyl acetate were acquired from Burdick & Jackson Inc. (Muskegon, MI, USA). Sodium chloride and sodium sulfate were purchased from Junsei Chemical Co., Ltd. (Tokyo, Japan).

### Soil and cucumber samples

Soil used for the indoor tests was collected from the top soil (0–15 cm depth) of an upland bean field located in Gunwi (GW), Republic of Korea. This filed which is a private land of Kyungpook National University in Republic of Korea was used for our experiment under prior approval. The collected soil was immediately dried in the shade and passed through a 2-mm sieve. Properties of the GW soil such as pH, organic matter (OM) content, exchangeable cations, electrical conductivity, cation exchange capacity (CEC), and particle distribution were measured using the official soil analysis methods established by the Rural Development Administration (RDA) of Republic of Korea (Table 1) [14].

**Table 1:** Physicochemical properties of the tested soils.

			Exchangeable cations (cmol_c_ kg^-1^)					Particle distribution (%)		
Sampling site	Soil texture	pH (1:5)	K	Ca	Mg	OM^a^ (g kg^-1^)	EC^b^ (dS m^-1^)	Sand	Silt	Clay
GW^c^	Sandy loam	5.6 ± 0.1	1.0 ± 0.0	3.0 ± 0.1	0.3 ± 0.0	17.2 ± 0.5	8.8 ± 0.2	58.3 ± 0.7	33.7 ± 1.2	8.0 ± 0.9
WG^d^	Sandy loam	6.3 ± 0.1	1.4 ± 0.6	16.4 ± 0.2	4.3 ± 0.4	37.0 ± 1.3	9.2 ± 0.4	55.0 ± 2.4	40.3 ± 0.8	4.7 ± 0.4

^a^OM, organic matter;

^b^EC, electrical conductivity;

^c^GW, soil sampled in the Gunwi region

^d^WG, soil sampled in the Waegwan region

One-month-old cucumber plug seedlings of the Nakdong-Banjjaki cultivar were purchased from a professional plant nursery located in Changnyeong, Republic of Korea, immediately transported to the laboratory, and placed in a growth chamber (JSPG-1500C, JS Research Inc., Republic of Korea) set at 26 ± 2°C, 75 ± 5% humidity, 30,000 lux light, and 12 h:12 h day:night photoperiod. They were provided with adequate water. After 3 d in the growth chamber, the cucumbers were sorted into similar sizes, and then assigned to the indoor or outdoor experiments.

### Indoor test

A mixed standard solution of α- and β-isomers in acetone was used to treat the GW soil at concentrations of 20 and 40 mg kg^-1^. The treated soils were repeatedly shaken until they were uniformly blended, and placed in a fume hood for 1 h to volatilize the acetone. The homogeneity of the treated soils was verified with recoveries of > 95% in the preliminary analysis of pesticide residue. For each concentration, 200 g of treated soil was placed in plastic pots (7.5 × 7.5 × 7.5 cm) lined with filter papers (90 mm i.d., Whatman No. 2, UK) at the bottom. Cucumber plug seedlings were transplanted into the pots (n = 20), and the pots were placed in the growth chamber under the same conditions as during adaptation period. During this experimental period, approximately 100 mL of water was provided to each pot every 12 h.

After the 15 and 30 d of growth, pots were taken from the chamber, and cucumber plants were divided into four compartments: roots, stems, leaves, and fruits. The weight and length of each plant compartment were measured. In the case of leaves, the number produced was counted instead of length. For roots, the length of the longest primary root was recorded as length. Before measuring root weight, root parts were washed in running water to remove attached soil particles, and lightly wiped with paper towels. Each compartment of the cucumber plants was individually homogenized using a grinder and stored in a -20°C freezer (GC-124HGFP, LG Electronics Inc., Republic of Korea) prior to pesticide residue analysis. Soil samples collected from the pots were air-dried in the shade for 5 d, passed through a 2-mm sieve, and stored in a -20°C freezer. Controls consisting of either pots (*n* = 20) with treated soils but no seedlings or pots (*n* = 20) with seedlings but no pesticide treatment were analyzed at the same time as treatment groups using the methods described above.

### Outdoor test

The outdoor tests were conducted in a greenhouse (2,160 m^2^ in area) on a cucumber farm located in Waegwan (WG), Republic of Korea, between February 2 and June 2, 2014. Commercial ED (35% EC) diluted with 2 L of water was sprayed on experimental plots (*n* = 3) of WG soil (30 × 100 cm) using a shoulder-type compression sprayer, equipped with a 1-mm of nozzle (KS-10-3, Kwang Sung Co., Republic of Korea). The plots were treated with ED concentrations of approximately 20 and 40 mg kg^-1^. The treated soils were homogenized up to a depth of 10 cm and aged for 12 h before the cucumber plug seedlings were transplanted. The seedlings were placed in the treated soil at intervals of 40 cm, and the soil surface was covered with plastic mulching films. No additional pesticides were sprayed throughout the experimental period. Water was supplied to the seedlings at a flow rate of 1.0 L/h for 4 h every 3 d using a drip irrigation system. Conditions in the greenhouse were controlled at 24.9 ± 1.16°C and 63.6 ± 2.37% humidity.

Soil samples were collected from each experimental plot at 0 (3 h), 30, 60, 90, and 120 d after pesticide treatment, air-dried for 5 d, and passed through a 2-mm sieve. A portion of the samples was used for the soil property (Table 1) and water content analysis. The water content of soil samples was measured by comparing weight change before and after oven-drying for 24 h. Cucumber fruits were consecutively harvested from plants in each experimental plot at 60, 70, 90, 100, 110, and 120 d after pesticide treatment, and whole cucumber plants were collected at the end of the 120-d growth period. Separation, growth measurement, and homogenization of cucumber compartments were conducted as described for the indoor test. All prepared samples were stored at -20°C until the pesticide residue analysis.

### Pesticide residue analysis

Air-dried soil and cucumber samples, weighing 10 g each, were placed in 250 mL Erlenmeyer flasks, and 80 mL of acetone was added. Soil samples were shaken at 200 rpm for 30 min using a shaking incubator (Vision Scientific Co., LTD., Republic of Korea). Cucumber samples were homogenized at 12,000 rpm for 3 min using a homogenizer (AM-7, Nihonseiki Kaisha LTD., Japan). The extracts from both soil and cucumber samples were filtered through a Büchner funnel, and transferred to a separatory funnel containing 500 mL of water and 50 mL of saturated sodium chloride solution. The funnel was shaken vigorously with 50 mL of methylene chloride, and the organic solvent fraction was collected after passing it through sodium sulfate. The remaining water layer in the funnel was subjected to the same extraction step with 50 mL of methylene chloride. The organic solvent fractions were concentrated using a rotary vacuum evaporator (Laborota-4000, Heidolph Instrument GmbH & Co., Germany).

The concentrate was dissolved in 10 mL of *n*-hexane, and poured into a glass column (16 mm internal diameter [I.D.], 30 cm height) containing 10 g of florisil and 3 g of sodium sulfate. The eluting solvents used for the clean-up were 60 mL of *n*-hexane to discard and 70 mL of ethyl acetate/*n*-hexane (20/80, v/v) to collect. The eluate was concentrated and dissolved in 2 mL of acetone. A 1-μL aliquot of final solution was injected into a gas chromatography-mass spectrometer (GC-MS; Shimadzu GC 2010 equipped with a GC-MS QP-2010 Plus, Kyoto, Japan). Residual amounts of ED isomers and their metabolite in samples were calculated using the regression equation obtained from matrix matched calibration (MMC) curves. Concentrations of ED residues were corrected as concentrations to oven-dried soil, using the soil water contents measured at each sampling for soil samples.

All graphs were created using the program SigmaPlot 10.0 ver., and degradation half-lives (DT_50_) of the ED isomers were calculated using Eq 1

$${\text{DT}}_{50}=\text{ ln}\left(2\right)/k$$ (1)

where *k* is degradation constant.

### Instrumental conditions

The temperatures of the GC-MS were set at 260°C for the injection port, 300°C for the interface, and 200°C for the ion source box. A oven, equipped with a Zebron ZB-SemiVolatiles capillary GC column (30 m length × 0.25 mm I.D. × 0.25 μm film thickness; Phenomenex, Tprrance, CA), was initially maintained at 100°C for 2 min, then increased by 280°C at the rate of 10°C min^-1^, and finally maintained at that temperature for 6 min. The target ions for selected ion monitoring (SIM) analysis were m/z 241 and 339 for the ED isomers, and m/z 272 and 387 for the ED-sulfate.

### Quality control

To perform recovery tests, a mixed standard solution containing α-, β-ED, and ED sulfate was present in the same ratio (1:1:1, w/w/w) and was prepared at the concentration of 10 μg mL^-1^ in acetone. The prepared mixture solution was added into each blank sample, which contained no ED compounds to achieve levels of 0.2 and 1.0 μg g^-1^, and these samples were subjected, in triplicate, to the analysis of ED residues using the previously described analytical method. Blank samples without ED spiking were prepared as quality control (QC) samples to identify ED compounds from the matrix. Similarly, blank samples were used in the preparation of MMC solution. To obtain the MMC solution, the blank samples were filled with ED mixture solution prepared at known concentrations. Seven concentration levels of pesticides were applied for the calibration: 0.1, 0.2, 0.5, 1.0, 2.0, 5.0, and 10.0 μg mL^-1^. The linearity of MMC curves was confirmed during every sample analysis, and the limits of quantitation (LOQs) for each sample were calculated based on the minimum detectable amount (MDA) of ED compounds in the GC-MS instrument.

## Results and Discussion

### Pesticide residue analysis

Total ion chromatogram and mass spectra of α-, β-isomers, and sulfate-metabolite of ED identified by GC-MS are shown in S1 Fig Fragment ions of each compound present in the mass spectra corresponded to the mass spectral library data provided by the National Institute of Standards and Technology (NITS), having similarities > 92%. Of the fragment ions, target ions used for the SIM analysis were the most intensive and free from fragment ions that appeared in blank samples of soil and cucumber compartments. The GC-MS chromatograms analyzed in the SIM mode using target ions for compounds are shown in S2 and S3 Figs Peaks of ED residues in the samples spiked with 1.0 mg kg^-1^ concentration exhibited good shape and selectivity. In addition, there were no interfering substances in the analysis of ED residues in cucumber compartments or soil samples. Correlation coefficients of calibration curves of the ED isomers and their metabolite were acceptable > 0.998. As shown in S1 Table, recovery rates of ED isomers and their metabolite in each sample obtained from indoor and outdoor tests were satisfactory between 85.4 and 103.9%, and coefficients of variation (CVs) ranged from 1.0 to 15.4%. In addition, the MDA and LOQ of the ED isomers and their metabolite were 0.1 ng and 0.02 mg kg^-1^, respectively.

### Growth of cucumber plants

Changes in the weights and lengths of cucumber compartments grown in the indoor test (S2 Fig) were associated with coefficients of variation (CV) of less than 20.1% and 38.6%, respectively. The lengths of cucumber compartments increased steadily throughout the indoor growth period (S4a Fig), while their weights doubled during the first 15 d, and then remained relatively constant (S4b Fig). Consequently, length and weight changes were not correlated in the indoor test. In contrast, in the outdoor test, weights and lengths of cucumber compartments were measured just once at the end of the experiment (S2 Table).

### Indoor test

#### ED residue in soil

ED residues in soils collected in the indoor test are presented in S3 Table. The initial concentrations of α- and β-isomers of ED in pots treated with 20 mg kg^-1^ of pesticide were found to be 12.7 and 5.7 mg kg^-1^, respectively. In the pots treated with 40 mg kg^-1^ concentration, the initial residual amounts were 18.7 and 9.0 mg kg^-1^ for α- and β-isomers, respectively. In this treatment pot, the residual amount summing both the isomer residues was 27.7 mg kg^-1^ and lower than the 40 mg kg^-1^ concentration. However, ED concentration of 27.7 mg kg^-1^ was high enough to determine the concentration dependency of pesticide uptake in cucumbers.

Alpha- and β-isomers in soils which cucumbers were grown decreased by 74.2% (DT_50_ = 15.2 d) and 46.3% (DT_50_ = 33.6 d), respectively, after 30 d of cultivation in the 20 mg kg^-1^-treated pots. However, in pots treated with 40 mg kg^-1^ concentration, α- and β-isomers decreased by 59.8 (DT_50_ = 22.8 d) and 36.1% (DT_50_ = 46.5 d), respectively. These results suggest that the degradation rates of isomers in soil are related to the application concentration as well as the type of ED isomer. Our findings are consistent with other studies that have reported higher degradation rates of the α-isomer in soil than the β-isomer, and have attributed this difference to the greater conversion potential of the α-isomer to ED-sulfate [15,16]. During the experimental period, 2.1 mg kg^-1^ of the ED-sulfate in soils was produced in the 20 mg kg^-1^-treated pots, and 2.8 mg kg^-1^ in 40 mg kg^-1^-treated pots. DT_50_ values of total ED (sum of isomers and metabolite) in the 20 and 40 mg kg^-1^-treated soils were 26.7 and 38.1 d, respectively. The half-lives of total ED obtained in our study were shorter than those reported in previous studies [17,18].

In contrast, the DT_50_ values of α- and β-isomers in control soils, in which cucumbers were not grown, were 13.9 and 23.9 d, respectively, in the 20 mg kg^-1^-treated pots, and 18.6 and 31.8 d, respectively, in the 40 mg kg^-1^-treated pots. Overall, the degradation rates of the ED residues in control soils were higher than those in cucumber-cultivated soils.

#### ED residue in cucumber

**Fig 1:**
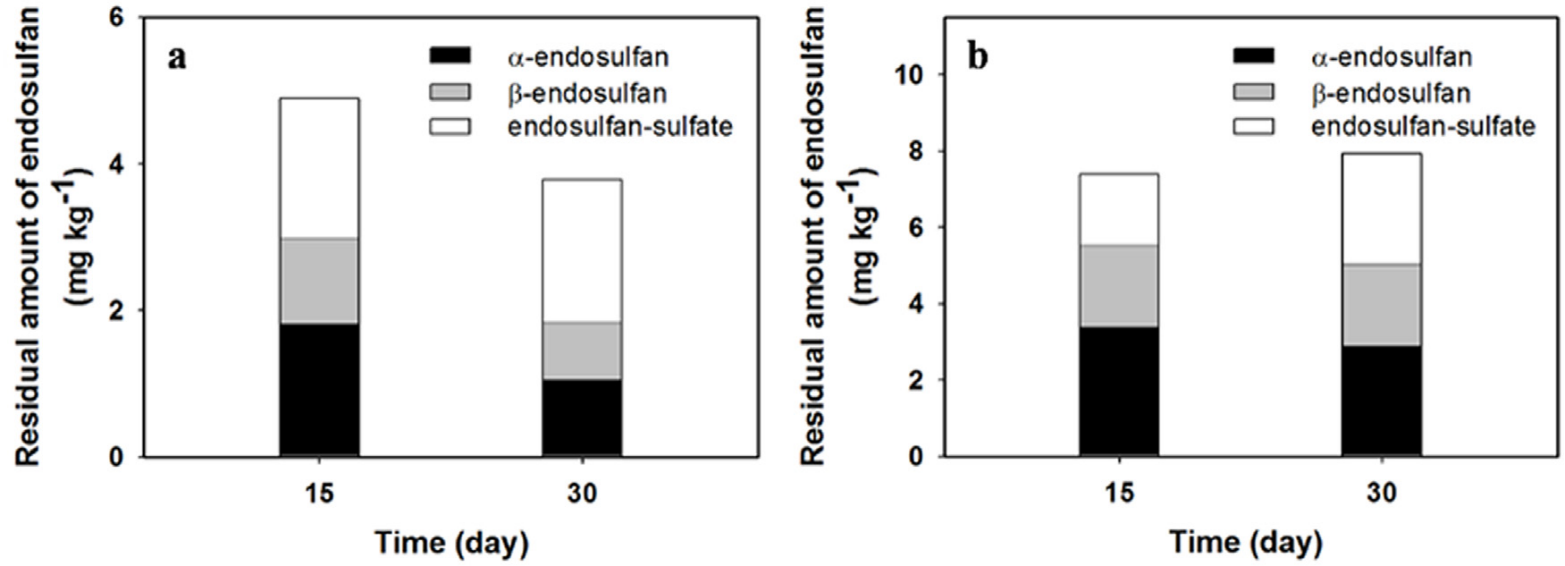
Uptake patterns of isomers and sulfate-metabolite of ED from soils in cucumber grown under two treatment concentrations: (a) 20 mg kg^-1^ and (b) 40 mg kg^-1^ in an indoor test.

In the 20 mg kg^-1^-treated pots, the uptake rates of the isomers in cucumbers sampled after 15 d of growth were 0.6% of the initial soil residue for the α-isomer, and 0.9% for the β-isomer (Fig 1a). However, after 30 d of growth, uptake rates decreased to 0.4% and 0.6% for α- and β-isomers, respectively (Fig 1a). The overall reduction in uptake amounts by the end of the growth period may be due to dilution by plant growth and simultaneous degradation of pesticides in both soil and plants [3]. The uptake rate of ED-sulfate in pesticide-treated pots after 15 d of growth was 4.6% of the total amount of the metabolite produced and decreased to 4.1% after 30 d.

In the 40 mg kg^-1^-treated pots, the uptake amounts of isomers in cucumber were considerably higher than those observed in the 20 mg kg^-1^-treated pots (Fig 1b). After 15 d of growth, uptake rates of α- and β-isomers relative to initial soil residue amount were 0.8 and 1.1%, respectively, and then remained constant throughout the experimental period. Of the ED-sulfate produced in the 40 mg kg^-1^-treated pots, 3.3% was absorbed by plants after 15 d, and this residual amount increased to 4.6% by end of 30 d.

Overall, these results indicate that the ED residue most absorbed in the cucumbers was ED-sulfate in the 20 mg kg^-1^-treated plots and the α-isomer in the 40 mg kg^-1^-treated plots. Singh and Singh [19] reported that the dominant ED residue absorbed from soils by various plants was β-isomer, which was contrary to the findings of the present study. In our study, although the β-isomer was detected in the smallest amount in cucumbers in all the treatment plots, its uptake rate was approximately 1.5-fold higher than that of the α-isomer. The greater uptake rate of β-isomer by plants may be the result of the greater aqueous solubility of the β-isomer than the α-isomer (~10-fold) [11]. This may also result from a slower dissipation rate of β-isomer driven by lower Henry’s law constant, vapor pressure, and volatility [20,21].

**Fig 2:**
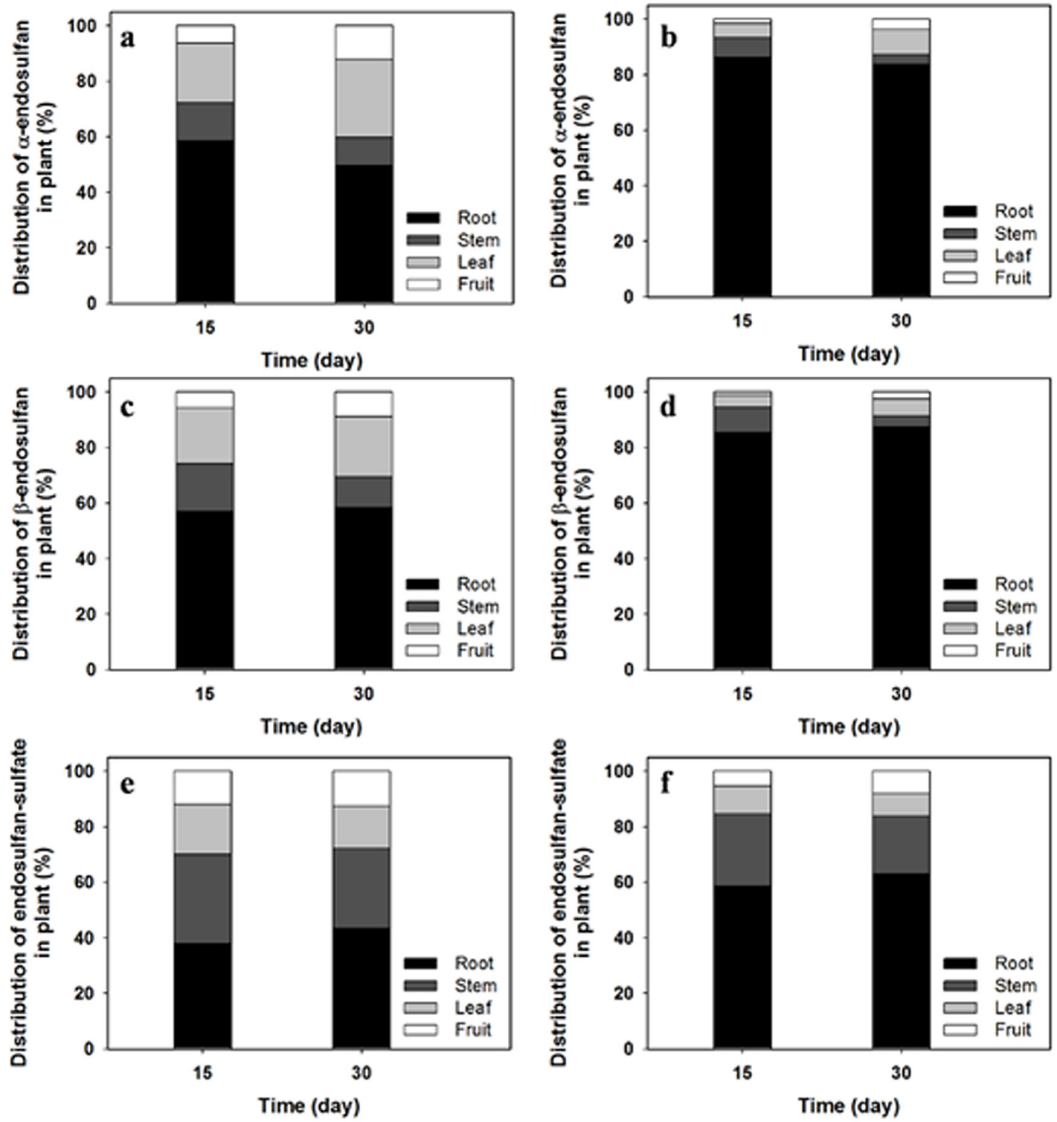
Time-dependent distribution of α- (a, b) and β-isomers of ED (c, d), as well as its sulfate-metabolite (e, f), in each cucumber compartment under treatment concentrations of 20 mg kg^-1^ (a, c, e) and 40 mg kg^-1^ (b, d, f) as observed in the indoor test.

The distribution of the ED residues absorbed from soil in various compartments of cucumber plants is described in Fig 2. In the 20 mg kg^-1^-treated pots, the highest amounts of the isomer residues were present in the roots, followed by the leaves, stems, and fruits. In the 40 mg kg^-1^-treated pots, over 83% of the absorbed isomers were located in the roots throughout the experimental period; other compartments contained only minor amounts of residue. ED-sulfate was also highest in the roots, and the remainder was found in the following order: stems > leaves > fruits. Total ED in edible fruit parts was considerably high and was in the range of 0.6–2.5 mg kg^-1^ (S3 Table). Although there are few studies on the uptake and distribution of ED residues in plants, studies on the uptake and distribution of other organochlorine pesticides from soils in various plant compartments [22,23], contrary to our findings, reported that the highest residual amounts were found in the leaves, followed by stems and roots. However, Esteve-Turrillas *et al*. [24] reported that in lettuce, greater residues of ED were found in the roots than in the leaves, a pattern similar to that reported herein.

### Outdoor test

#### ED residue in soil

Initial concentrations of α- and β-isomers in soils, collected in the outdoor test, were 18.3 and 9.2 mg kg^-1^, respectively, in 20 mg kg^-1^-treated plots and 33.7 and 16.1 mg kg^-1^, respectively, in 40 mg kg^-1^-treated plots. The sum of these initial concentrations of isomers in 20 and 40 mg kg^-1^-treated plots were 27.5 and 49.8 mg kg^-1^, respectively, and were slightly higher than the concentrations.

**Fig 3:**
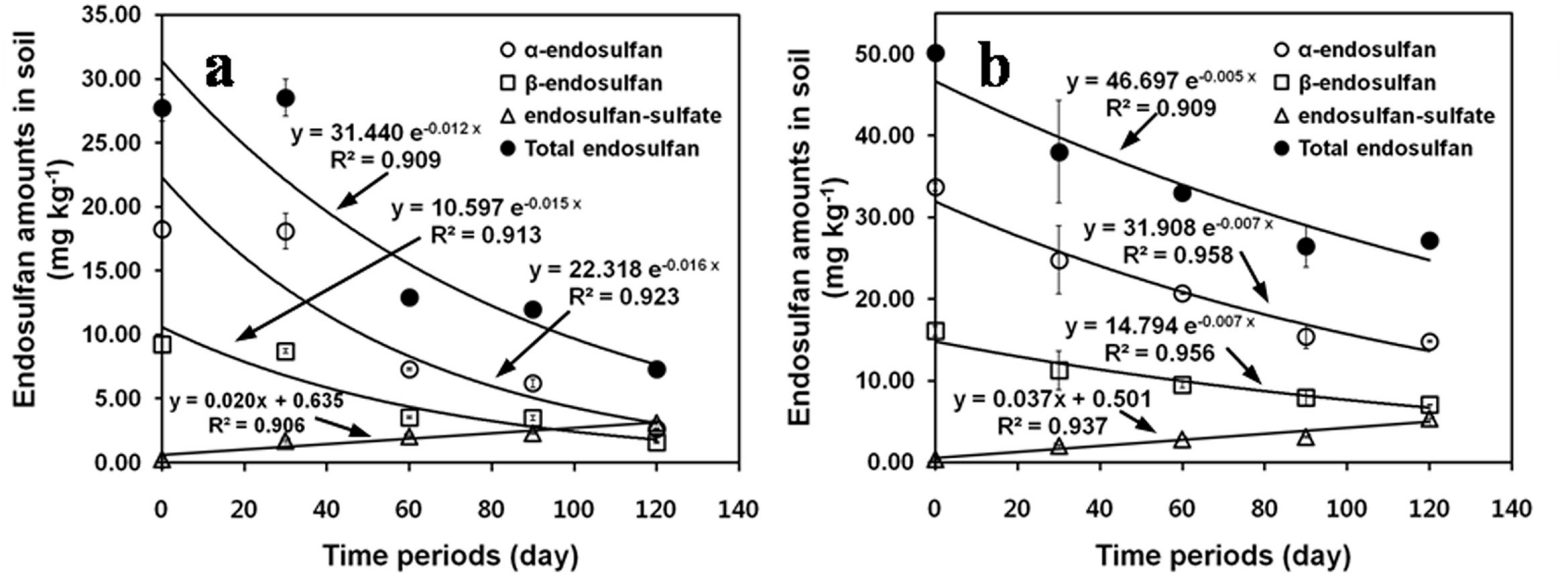
Residual patterns of isomers and sulfate-metabolite of ED in soils in (a) 20 mg kg^-1^ and (b) 40 mg kg^-1^-treated plots as observed in an outdoor greenhouse experiment.

In the 20 mg kg^-1^-treated plots, DT_50_ values of α- and β-isomers were 42.0 d and 46.8 d, respectively (Fig 3a). However, DT_50_ values of the isomers in the 40 mg kg^-1^-treated plots were 97.6 and 105.0 d for the α- and β-isomers, respectively, and 2-flod longer than those in the 20 mg kg^-1^-treatment plots (Fig 3b). All the DT_50_ values of isomers observed in the outdoor test corresponded closely with values reported in our indoor study (i.e., 7–75 d for the α-isomer and 33–376 d for the β-isomer) [11]. At the end of the 120 d growth period in the greenhouse, ED-sulfate produced in soils was 3.1 mg kg^-1^ in the 20 mg kg^-1^-treated plots and 5.3 mg kg^-1^ in the 40 mg kg^-1^-treated plots (Fig 3). DT_50_ values of total ED in soils were 52.1 d in the 20 mg kg^-1^-treated plots and 115.5 d in the 40 mg kg^-1^-treated plots.

The overall degradation rates of ED residues in soils were markedly lower in the outdoor test than in the indoor test. These differences between the degradation rates in the indoor and outdoor tests might be the result of soil preparations, such as sieving and drying, which were used in the indoor test but not in the outdoor test. Furthermore, soil microbes can also influence the ED biodegradation rates, leading to differences in degradation rates [25]. For example, Lee *et al*. [26] found that in soils with microbes the ED isomers were degraded by 52% within 7 d of incubation, and Shivaramaiah and Kennedy [27] found they degraded by 50% within 3 d of incubation.

#### ED residue in cucumber

**Fig 4:**
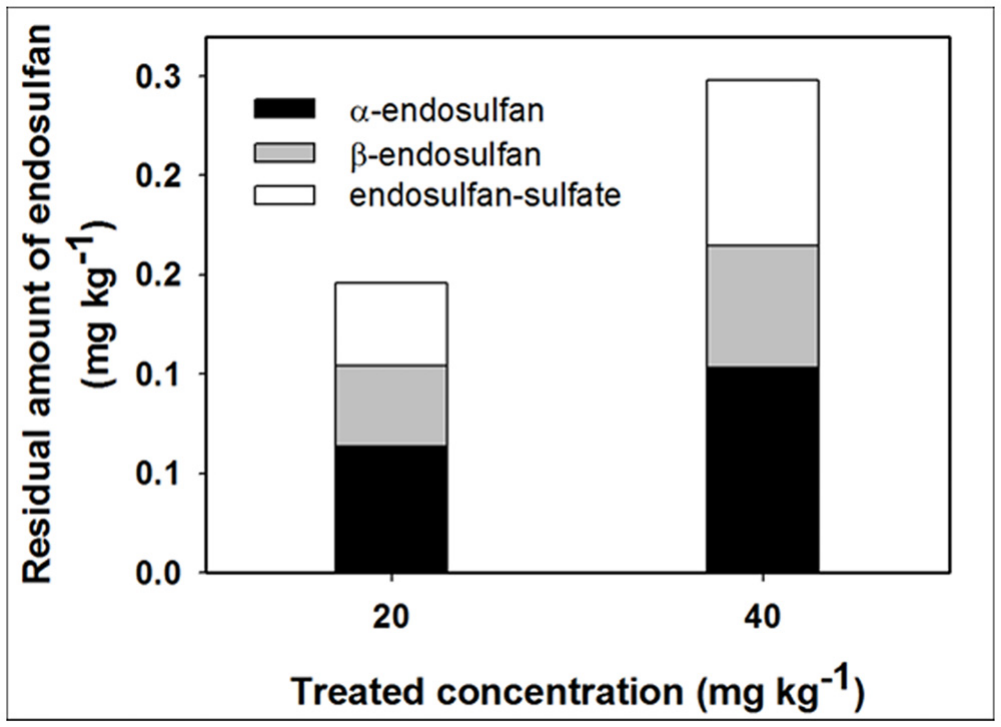
Distribution patterns of isomers and sulfate-metabolite of ED absorbed from the soil into cucumbers grown for 120 d under greenhouse conditions.

At the end of the 120 d growth period, uptake rates of the isomers into cucumber, relative to the initial soil residue amount, in both 20 and 40 mg kg^-1^-treated plots were 0.02% for the α-isomer and 0.01% for β-isomer. The amount of the ED-sulfate absorbed in 20 and 40 mg kg^-1^-treated plots was 0.12 and 0.14% of that produced in the soil, respectively. According to these results, although absolute amounts of ED and its metabolite in a cucumber plant increased with increasing treatment concentrations (Fig 4), uptake rates were similar at all treatment concentrations. This trend was observed in the indoor tests as well (Fig 1).

The α-isomer was the most dominant isomer in the cucumber in the outdoor experiment (Table 2). In addition, the most ED isomers in the outdoor test were found in the roots (> 80%), followed by the leaves (6.1–12.8%) and stems (2.2–8.0%); the uptake amount of ED-sulfate by the cucumber occurred in the following order: root (79.3–87.8%) > stems (11.2–12.2%) > leaves (0.0–9.5%). In contrast with results of the indoor test, the residual amounts of ED in all fruits harvested during the outdoor test were less than LOQ (0.02 mg kg^-1^). While there is no set maximum residue limit (MRL) for ED in cucumber fruits in Korea, the MRL in the codex criterion was set at 1.0 mg kg^-1^ [28]. Our results indicate that if the residual amount of total ED in agricultural soils is less than 49.8 mg kg^-1^, then the uptake amount transferred from soil to cucumber fruits might be below the codex MRL.

**Table 2:** Residue amounts of the isomers and sulfate-metabolite of ED in various compartments of cucumber plants grown for 120 d under greenhouse conditions.

		Residual amount^a^ (mg kg^-1^)				
		Cucumber part				
ED type	Treatment level (mg kg^-1^)	Leaf	Stem	Root	Fruit	Whole
Alpha-	20	0.04 ± 0.005	< LOQ^b^	1.68 ± 0.067	< LOQ^b^	0.06 ± 0.002
	40	0.16 ± 0.026	< LOQ^b^	2.76 ± 0.112	< LOQ^b^	0.10 ± 0.005
Beta-	20	0.04 ± 0.006	0.02 ± 0.011	0.97 ± 0.052	< LOQ^b^	0.04 ± 0.003
	40	0.08 ± 0.017	0.02 ± 0.008	1.63 ± 0.069	< LOQ^b^	0.06 ± 0.003
-sulfate	20	< LOQ^b^	0.03 ± 0.010	1.05 ± 0.056	< LOQ^b^	0.04 ± 0.003
	40	0.10 ± 0.011	0.06 ± 0.001	2.08 ± 0.019	< LOQ^b^	0.08 ± 0.001
Total	20	0.09 ± 0.009	0.06 ± 0.021	3.69 ± 0.172	< LOQ^b^	0.15 ± 0.007
	40	0.34 ± 0.026	0.07 ± 0.008	6.47 ± 0.166	< LOQ^b^	0.25 ± 0.007

^a^Mean of triplication ± SD

^b^Less than the limit of quantification (LOQ), 0.02 mg kg^-1^

In the present study, overall patterns of uptake and distribution of the ED residues in cucumbers, obtained in the indoor and outdoor tests were similar; however, the absolute uptake amounts between the tests were not comparable due to different overall growth periods. Furthermore, the similarity in the residual patterns of ED in cucumber between the indoor and outdoor tests suggests that the use of indoor tests to describe the plant uptake pattern of pesticide in agricultural fields may have some benefit. However, various species of plants differ in their uptake patterns. For example, Singh and Singh [19] found that the accumulation patterns of ED in each part of seven wild plants, growing in contaminated land, varied depending on plant species. Therefore, further studies based on both indoor and outdoor experiments are required to understand patterns of pesticide uptake in various plants in order to obtain safer agricultural products from soil persistent pesticides.

## Supporting Information

S1 Fig Total ion chromatogram and mass spectra of α- (a), β- (b) isomers and sulfate-metabolite (c) of ED analyzed using GC-MS. (TIF)

S2 Fig Chromatograms for recovery tests of α- and β-isomers and sulfate-metabolite of ED with 1.0 mg kg-1-spiked concentration in whole plants of cucumbers cultivated during non-ED amended indoor (a, b) and outdoor (c, d) tests. (a, c—spiked samples; b, d—control samples). (TIF)

S3 Fig Chromatograms for recovery tests of α-, β-isomers and sulfate-metabolite of ED with 1.0 mg kg^-1^-spiked concentration in non-ED amended soils during indoor (a, b) and outdoor (c, d) tests. (a, c—spiked samples; b, d—control samples) (TIF)

S4 Fig Change in length (a) and weight (b) of cucumber parts during the indoor test. (TIF)

S1 Table Recovery rates of ED isomers and their sulfate metabolite in soil and each cucumber compartment. (DOCX)

S2 Table Lengths and weights of cucumber parts cultivated for 120 days in the greenhouse. (DOCX)

S3 Table Time-dependent residual amount of ED isomers and its metabolite in cucumber plants cultivated on the artificially treated soil under growth chamber conditions. (DOCX)
