# Supplementary figures and images for: Plant Uptake and Distribution of Endosulfan and Its Sulfate Metabolite Persisted in Soil
Source: PLoS One. 2015 Nov 3;10(11):e0141728. doi: 10.1371/journal.pone.0141728 (PMC4631486; doi:10.1371/journal.pone.0141728)

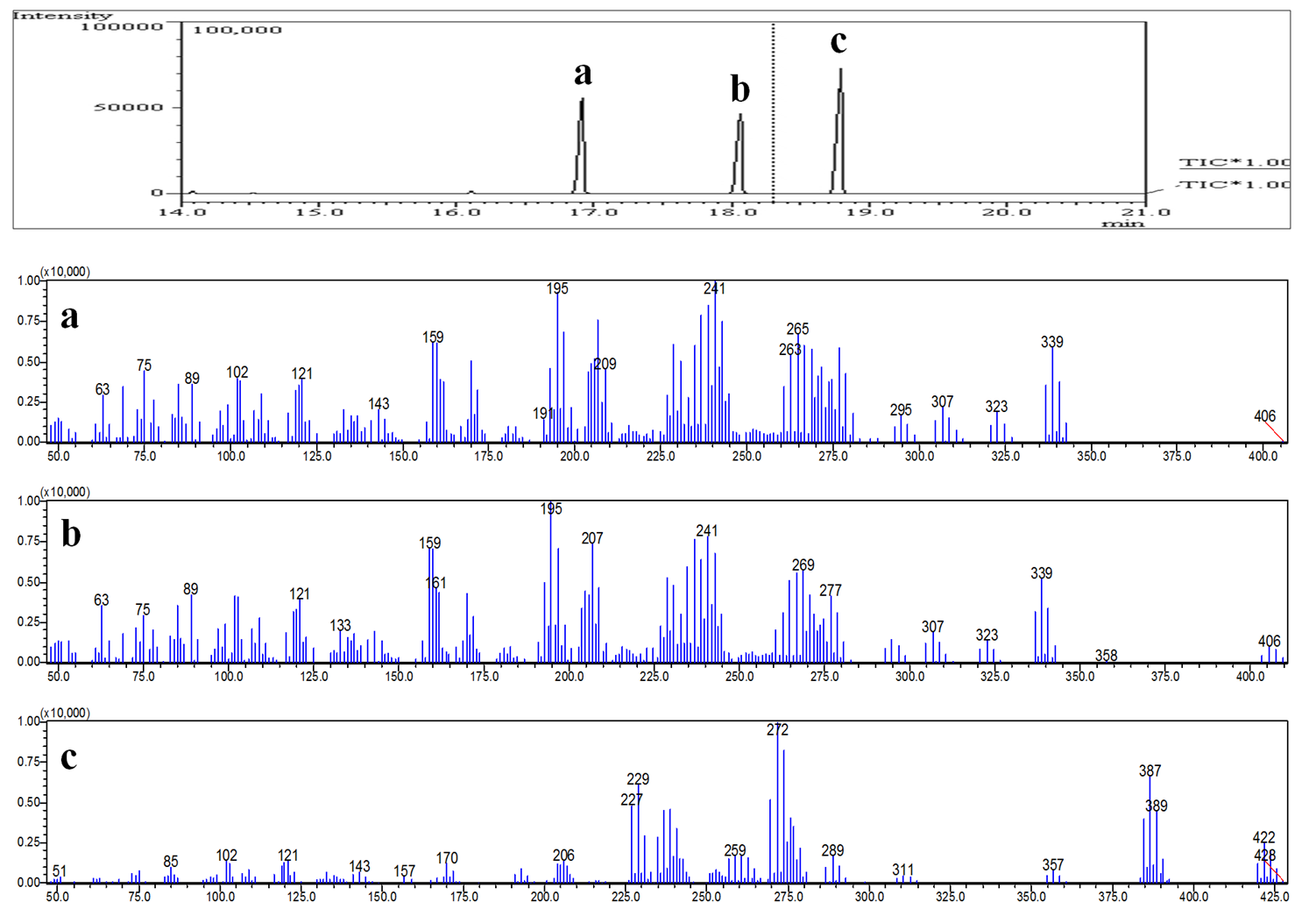

Supplement: S1 Fig — (TIF) [file pone.0141728.s001.tif]

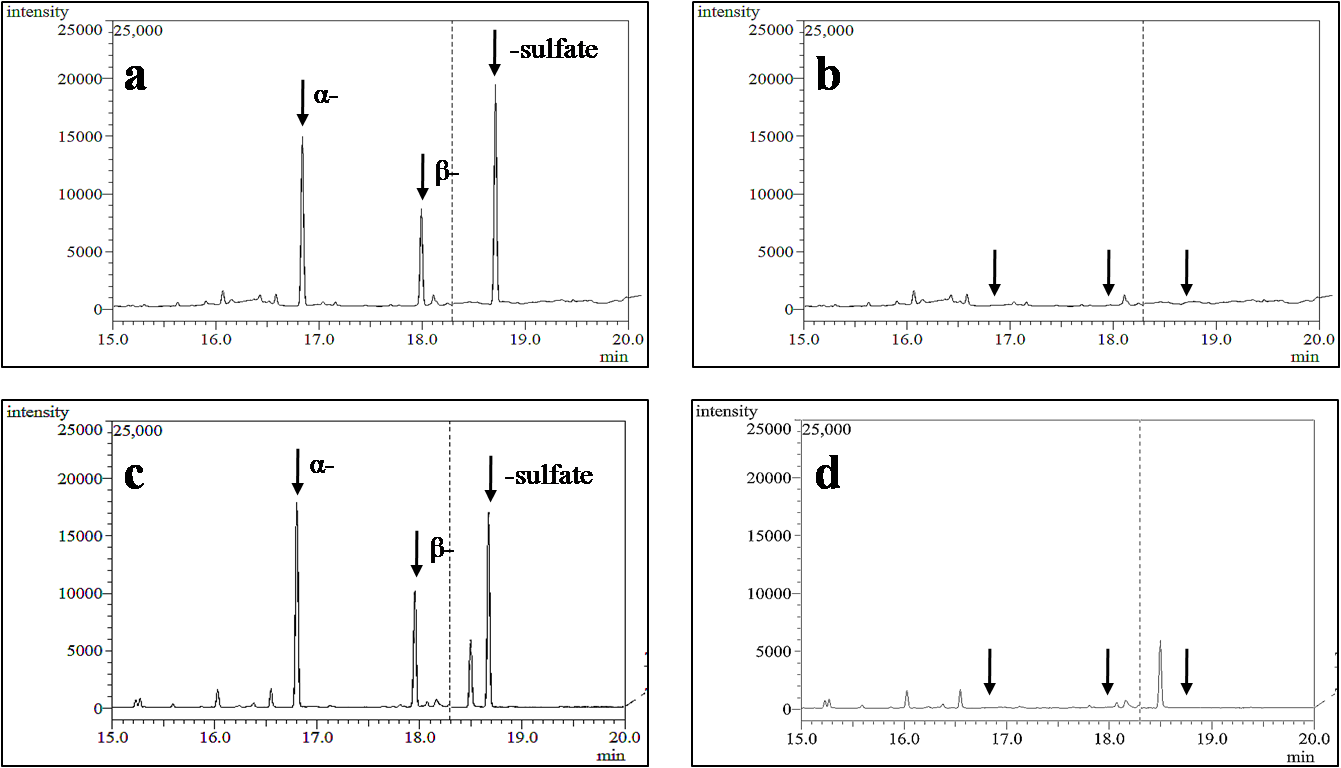

Supplement: S2 Fig — (TIF) [file pone.0141728.s002.tif]

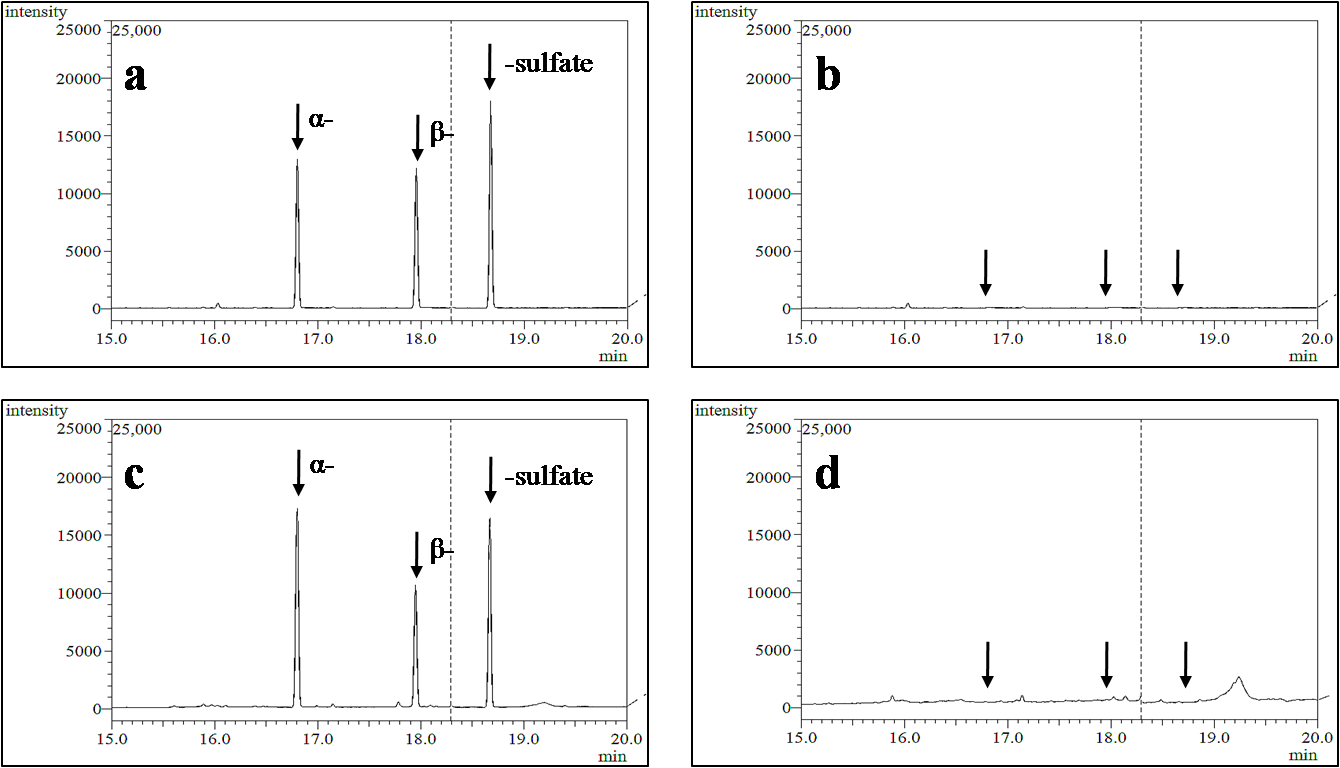

Supplement: S3 Fig — (TIF) [file pone.0141728.s003.tif]

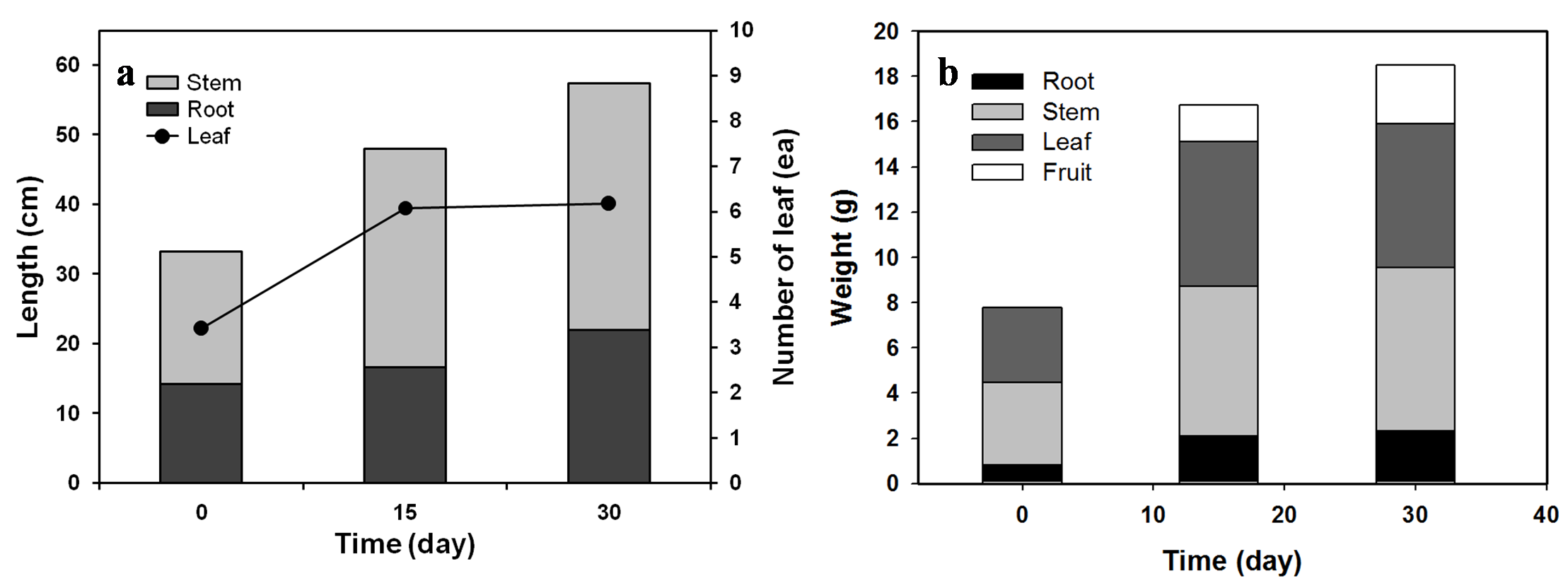

Supplement: S4 Fig — (TIF) [file pone.0141728.s004.tif]
