# Supplementary material for: Plant Uptake and Distribution of Endosulfan and Its Sulfate Metabolite Persisted in Soil
Source: PLoS One. 2015 Nov 3;10(11):e0141728. doi: 10.1371/journal.pone.0141728 (PMC4631486; doi:10.1371/journal.pone.0141728)
Supplement: S1 Table — (DOCX) [file pone.0141728.s005.docx]

Table S1. Recovery rates of ED isomers and their sulfate metabolite in soil and each cucumber compartment

| Experimental type | Endosulfan  type | Treatment  level  (mg kg^-1^) | Recovery rate^a)^ (%) ± SD | | | | |
| --- | --- | --- | --- | --- | --- | --- | --- |
|  |  |  | Leaf | Stem | Root | Fruit | Soil |
| Indoor | Alpha- | 0.2 | 102.3±5.1 | 102.9±2.6 | 89.8±0.7 | 86.7±5.3 | 92.4±7.0 |
|  |  | 1.0 | 85.4±3.7 | 87.2±3.0 | 93.8±1.0 | 86.2±2.6 | 97.6±6.1 |
|  | Beta- | 0.2 | 92.9±4.3 | 102.5±2.3 | 87.7±0.9 | 88.7±7.4 | 87.6±6.7 |
|  |  | 1.0 | 89.7±3.4 | 87.7±2.5 | 92.1±1.1 | 93.6±4.2 | 95.8±6.8 |
|  | -sulfate | 0.2 | 86.2±9.1 | 87.6±2.6 | 89.1±0.9 | 102.5±10.9 | 92.6±5.2 |
|  |  | 1.0 | 85.4±3.7 | 95.6±1.6 | 98.8±0.9 | 88.6±9.0 | 103.9±8.8 |
| Outdoor | Alpha- | 0.2 | 86.3±7.0 | 85.9±2.5 | 81.1±1.4 | 100.9±11.2 | 85.4±0.9 |
|  |  | 1.0 | 87.4±5.2 | 94.1±4.2 | 87.4±3.2 | 95.1±10.3 | 100.2±7.3 |
|  | Beta- | 0.2 | 91.2±3.9 | 87.6±3.7 | 86.5±1.4 | 89.7±6.7 | 94.2±0.9 |
|  |  | 1.0 | 93.5±6.7 | 90.2±2.8 | 89.2±2.5 | 90.0±7.7 | 101.5±7.6 |
|  | -sulfate | 0.2 | 88.8±8.1 | 84.1±3.4 | 98.0±1.0 | 95.4±4.1 | 86.3±6.3 |
|  |  | 1.0 | 90.1±6.4 | 82.3±3.9 | 84.3±3.5 | 96.3±3.9 | 87.3±12.4 |

^a)^ Mean of triplication ± SD
