# Supplementary material for: Plant Uptake and Distribution of Endosulfan and Its Sulfate Metabolite Persisted in Soil
Source: PLoS One. 2015 Nov 3;10(11):e0141728. doi: 10.1371/journal.pone.0141728 (PMC4631486; doi:10.1371/journal.pone.0141728)
Supplement: S2 Table — (DOCX) [file pone.0141728.s006.docx]

Table S2. Lengths and weights of cucumber parts cultivated for 120 days in the greenhouse

| Item | Cucumber part | | | |
| --- | --- | --- | --- | --- |
|  | Leaf | Stem | Root | Fruit^b)^ |
| Length (cm) | 16 ± 2^a)^ | 393.1 ± 40.2 | 23.1 ± 6.7 | 25.2 ± 3.1 |
| Weight (g) | 94.8 ± 20.5 | 173.4 ± 29.6 | 36.3 ± 13.1 | 155.1 ± 24.2 |

^a)^ Unit as the number of leaves

^b)^ Average values for lengths and weights of fruits during whole periods of the outdoor test
