# Supplementary material for: Plant Uptake and Distribution of Endosulfan and Its Sulfate Metabolite Persisted in Soil
Source: PLoS One. 2015 Nov 3;10(11):e0141728. doi: 10.1371/journal.pone.0141728 (PMC4631486; doi:10.1371/journal.pone.0141728)
Supplement: S3 Table — (DOCX) [file pone.0141728.s007.docx]

**Table S3. Time-dependent residual amount of ED isomers and its metabolite in cucumber plants cultivated on the artificially treated soil under growth chamber conditions**

| Endosulfan  type | Treated level  (mg kg^-1^) | Passed time  (day) | Residual amount^a)^ (mg kg^-1^) | | | | | | | |
| --- | --- | --- | --- | --- | --- | --- | --- | --- | --- | --- |
|  |  |  | Cucumber part | | | | | Soil | | |
|  |  |  | Leaf | Stem | Root | Fruit | Whole | | Control^b)^ | Sample |
| Alpha- | 20 | 0 | -^c)^ | -^c)^ | -^c)^ | -^c)^ | -^c)^ | | 13.5 ± 0.57 | 12.7 ± 0.04 |
|  |  | 15 | 0.9 ± 0.15 | 0.6 ± 0.10 | 8.1 ± 0.31 | 0.6 ± 0.05 | 1.8 ± 0.05 | | 3.9 ± 0.15 | 4.2 ± 0.08 |
|  |  | 30 | 0.7 ± 0.70 | 0.2 ± 0.00 | 4.0 ± 0.10 | 0.7 ± 0.03 | 1.0 ± 0.02 | | 3.0 ± 0.21 | 3.3 ± 0.09 |
|  | 40 | 0 | -^c)^ | -^c)^ | -^c)^ | -^c)^ | -^c)^ | | 20.4 ± 0.35 | 18.7 ± 3.78 |
|  |  | 15 | 0.4 ± 0.05 | 0.5 ± 0.05 | 26.4 ± 0.86 | 0.2 ± 0.02 | 3.4 ± 0.14 | | 8.6 ± 0.07 | 10.0 ± 0.30 |
|  |  | 30 | 0.7 ± 0.00 | 0.2 ± 0.03 | 22.0 ± 0.46 | 0.4 ± 0.01 | 2.9 ± 0.05 | | 6.7 ± 0.04 | 7.5 ± 0.10 |
| Beta- | 20 | 0 | -^c)^ | -^c)^ | -^c)^ | -^c)^ | -^c)^ | | 5.9 ± 0.17 | 5.7 ± 0.04 |
|  |  | 15 | 0.6 ± 0.08 | 0.5 ± 0.07 | 5.1 ± 0.19 | 0.4 ± 0.05 | 1.2 ± 0.03 | | 3.1 ± 0.10 | 3.4 ± 0.06 |
|  |  | 30 | 0.4 ± 0.01 | 0.2 ± 0.01 | 3.5 ± 0.10 | 0.4 ± 0.02 | 0.8 ± 0.01 | | 2.5 ± 0.11 | 3.1 ± 0.08 |
|  | 40 | 0 | -^c)^ | -^c)^ | -^c)^ | -^c)^ | -^c)^ | | 9.8 ± 0.16 | 9.0 ± 1.93 |
|  |  | 15 | 0.2 ± 0.03 | 0.4 ± 0.05 | 16.6 ± 0.61 | 0.1 ± 0.01 | 2.1 ± 0.10 | | 5.9 ± 0.10 | 6.9 ± 0.17 |
|  |  | 30 | 0.3 ± 0.00 | 0.2 ± 0.01 | 17.1 ± 0.27 | 0.2 ± 0.01 | 2.1 ± 0.03 | | 5.1 ± 0.12 | 5.7 ± 0.15 |
| -sulfate | 20 | 0 | -^c)^ | -^c)^ | -^c)^ | -^c)^ | -^c)^ | | 0.0 ± 0.00 | 0.0 ± 0.00 |
|  |  | 15 | 0.8 ± 0.03 | 1.4 ± 0.09 | 5.5 ± 0.09 | 1.3 ± 0.03 | 1.9 ± 0.02 | | 1.8 ± 0.05 | 1.8 ± 0.03 |
|  |  | 30 | 0.7 ± 0.04 | 1.3 ± 0.02 | 6.5 ± 0.15 | 1.4 ± 0.02 | 2.0 ± 0.04 | | 2.0 ± 0.02 | 2.1 ± 0.08 |
|  | 40 | 0 | -^c)^ | -^c)^ | -^c)^ | -^c)^ | -^c)^ | | 0.0 ± 0.00 | 0.0 ± 0.00 |
|  |  | 15 | 0.5 ± 0.03 | 1.0 ± 0.10 | 9.8 ± 0.27 | 0.3 ± 0.02 | 1.9 ± 0.09 | | 2.3 ± 0.04 | 2.4 ± 0.06 |
|  |  | 30 | 0.6 ± 0.05 | 1.2 ± 0.05 | 16.7 ± 0.50 | 0.8 ± 0.05 | 2.9 ± 0.07 | | 2.7 ± 0.08 | 2.8 ± 0.06 |
| Total | 20 | 0 | -^c)^ | -^c)^ | -^c)^ | -^c)^ | -^c)^ | | 19.4 ± 0.73 | 18.4 ± 0.09 |
|  |  | 15 | 2.3 ± 0.25 | 2.4 ± 0.25 | 18.7 ± 0.57 | 2.3 ± 0.14 | 4.9 ± 0.07 | | 8.8 ± 0.21 | 9.4 ± 0.10 |
|  |  | 30 | 1.8 ± 0.07 | 1.7 ± 0.03 | 14.0 ± 0.29 | 2.5 ± 0.06 | 3.8 ± 0.07 | | 7.5 ± 0.28 | 8.4 ± 0.24 |
|  | 40 | 0 | -^c)^ | -^c)^ | -^c)^ | -^c)^ | -^c)^ | | 30.2 ± 0.51 | 27.7 ± 5.7 |
|  |  | 15 | 1.1 ± 0.11 | 1.9 ± 0.20 | 52.9 ± 1.73 | 0.6 ± 0.05 | 7.4 ± 0.32 | | 16.8 ± 0.20 | 19.3 ± 0.52 |
|  |  | 30 | 1.6 ± 0.06 | 1.6 ± 0.05 | 55.7 ± 1.21 | 1.4 ± 0.08 | 7.9 ± 0.15 | | 14.5 ± 0.23 | 16.0 ± 0.31 |

^a)^ Mean of triplication ± SD;

^b)^ Soil spiking only pesticide without cucumber plant; ^c)^ No experimental data
